# 

**Published:** 1882-01

**Authors:** 


ESTABLISHED IN 1855.

OLD SERIES.
Vol. XIX—No. 10.

JANUARY, 1882.

NEW SERIES.
Vol. I—No. 4.

### ATLANTA

### MEDICAL REGISTER.

(*ATLANTA MEDICAL AND SURGICAL JOURNAL.*)

EDITORS:

JNO. THAD. JOHNSON, M.D., JAMES B. BAIRD, M.D.

PUBLISHED BY H. H. DICKSON, AT $2.50 A YEAR IN ADVANCE.

ATLANTA, GEORGIA:

*H. H. DICKSON, BOOK AND JOB PRINTER AND BOOKBINDER.*

1882.

